# Mirizzi Syndrome: A Case Report

**DOI:** 10.7759/cureus.34783

**Published:** 2023-02-08

**Authors:** Bryce Grohol, Grayson T Fortin, Tyler Ingold, Paul Bennett

**Affiliations:** 1 Medicine, Liberty University College of Osteopathic Medicine, Lynchburg, USA; 2 Internal Medicine, Centra Lynchburg General Hospital, Lynchburg, USA

**Keywords:** gallbladder removal, common bile duct dilatation, cystic duct injury, gallstone disease (gsd), mirizzi syndrome

## Abstract

Mirizzi syndrome (MS) describes a rare complication of cholelithiasis resulting from extrinsic compression of the common hepatic duct by impacted gallstones in the cystic duct or Hartmann’s pouch. MS is most commonly seen in adults and is more prevalent in the female population. Due to the pathophysiology of MS being similar to other causes of cholecystitis and biliary obstruction, the symptomatology is rather nonspecific. While ultrasound and magnetic resonance cholangiopancreatography are commonly used for diagnosis, treatment of this condition typically involves cholecystectomy. Identifying MS versus other more common causes of obstructive jaundice is paramount in limiting complications. In this report, we describe a case of MS diagnosed in a 32-year-old male who presented with nonspecific abdominal pain and other signs of obstructive jaundice. The goal of this study is to show how identifying a rare underlying cause of a common presentation can lead to improved patient outcomes.

## Introduction

Mirizzi syndrome (MS) describes a rare complication of cholelithiasis leading to obstructive jaundice. The pathophysiology of the condition involves gallstones becoming impacted in either the cystic duct or in Hartmann’s pouch. These stones then produce a mass effect that extrinsically compresses the common hepatic duct resulting in the symptoms of obstructive jaundice [1-3]. First characterized in 1905 by German physician Kehr, and later named syndrome del conducto hepatico by Argentinian surgeon Pablo Mirizzi, MS often presents with nonspecific symptoms that can make the diagnosis challenging [2,3]. Given its unique presentation, early intervention is important to reduce further complications. MS typically peaks in late adulthood and appears to have a greater incidence in women compared to men [4]. The cited prevalence of MS is rare at approximately 1%, but in some underdeveloped countries in Latin America, the prevalence was documented as high as 5.7% [5,6]. While ultrasound is typically the first-line imaging study of choice, magnetic resonance cholangiopancreatography is also a highly effective and safe tool for working up patients suspected of MS. The mainstay treatment of MS most often involves cholecystectomy or subtotal cholecystectomy. While laparoscopy has its advantages, there is a high rate of conversion to open laparotomy which can result in high rates of complications [1]. Here we describe a case of MS diagnosed in a 32-year-old male who presented with nonspecific abdominal pain and other signs of obstructive jaundice.

## Case presentation

A 32-year-old male with a past medical history of irritable bowel syndrome and obesity presented to urgent care with chronic episodic epigastric pain radiating to his back and his right side. He described the pain as dull, however, periods of exacerbation were deep and gnawing. The patient described the pain as six out of 10 at baseline, however, exacerbations of the pain were up to a nine out of 10 at their worst. The patient did not believe the pain changed with his consumption of different foods. It typically lasted for approximately 48 hours and then temporarily alleviated until the next flare. He describes the pain as "a ball of discomfort at the base of my esophagus." He denied any fever, change in bowel habits, nausea, or vomiting.

Physical examination at urgent care showed a large abdomen with normoactive bowel sounds in all four quadrants. Mild epigastric pain was present, but the abdomen was otherwise nontender. There were no peritoneal signs, organomegaly, or costovertebral angle tenderness. Murphy's sign was negative. Radial and dorsalis pedis pulses were 2+ bilaterally. The skin was warm and dry without rash or lesions. There was no scleral icterus or change in weight. Vital signs were within normal limits and an electrocardiogram showed no acute cardiovascular changes. Due to a concern for gastritis, the patient’s esomeprazole was increased from 40 mg once daily to twice daily and Carafate was prescribed. The patient was instructed to go to the emergency department if he experienced any new or worsening symptoms.

The following day, the patient presented to the emergency department with worsening of his epigastric pain as well as new-onset nausea and vomiting. His labs returned with normal white blood cell count, hemoglobin, hematocrit, electrolytes, and nonelevated troponins. Initial liver function tests (LFTs) revealed total bilirubin of 4.7 mg/dL (normal range: 0.3-1.0 mg/dL), alkaline phosphatase (ALP) 156 U/L (normal range: 34-104 U/L), alanine aminotransferase (ALT) 1066 U/L (normal range: 7-52 U/L), and aspartate aminotransferase (AST) 1041 U/L (normal range: 13-39 U/L). The emergency physician at the time suspected transient common bile duct (CBD) obstruction and referred the patient to the hospitalist service for admission. Gastroenterology was also consulted.

**Figure 1:**
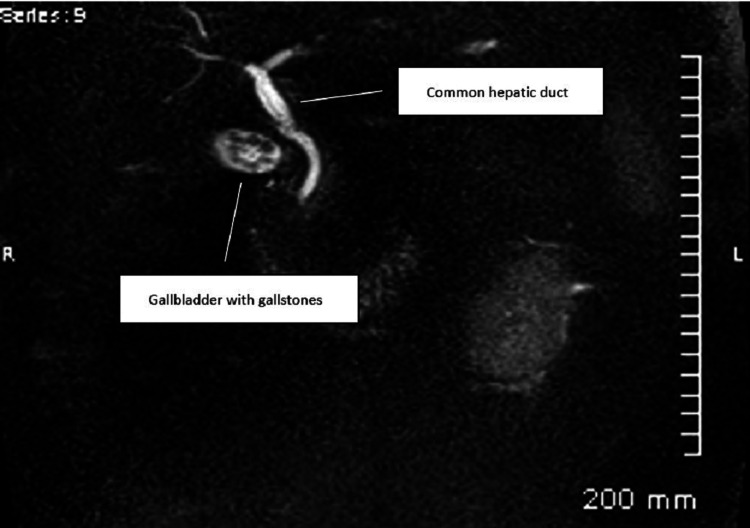
Patient's first MRCP on admission day one. MRCP: magnetic resonance cholangiopancreatography

Right upper quadrant (RUQ) gallbladder ultrasound later that day was limited due to body habitus and bowel gas. Fatty infiltration of the liver was noted, however, no other abnormalities were present including an absence of gallstones. Magnetic resonance cholangiopancreatography (MRCP) was completed the same day and showed a gallbladder with cholelithiasis present in the gallbladder neck, but showed no evidence of cholecystitis. There was no CBD dilatation, biliary strictures, or filling defects. The pancreas, spleen, kidney, and adrenals all were of normal size and signal intensity. The patient’s first MRCP is shown below in Figure 1.

On the second day of admission, gastroenterology discussed MS as a possibility for the first time. Due to endoscopic retrograde cholangiopancreatography (ERCP) not being able to be completed by the weekend physician, an endoscopic ultrasound (EUS) was performed to better visualize the biliary tree. LFTs were redrawn and showed a total bilirubin of 6.4 mg/dL, direct bilirubin of 4.6 mg/dL, ALP of 179 U/L, AST of 787 U/L, and ALT of 1182 U/L. Hepatitis serologies were negative.

**Figure 2:**
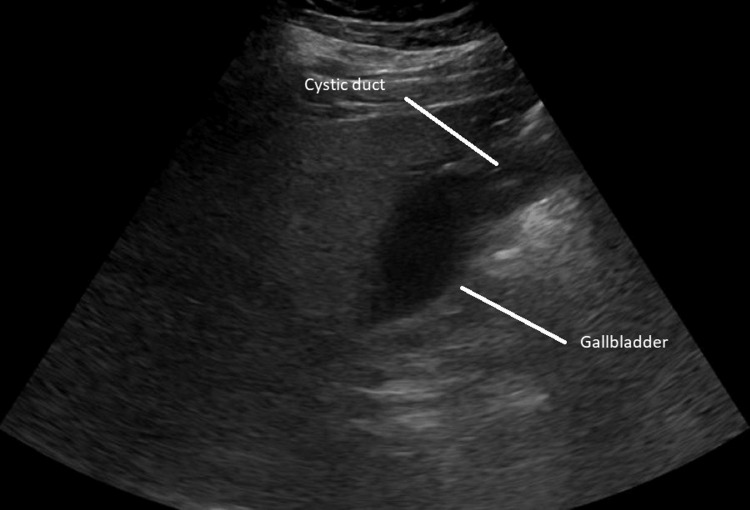
Endoscopic ultrasound completed on admission day two.

EUS showed no signs of ampulla abnormality and no masses, cysts, calcifications, or biliary sludging. Both the CBD and the common hepatic duct (CHD) showed dilatation. There were multiple small stones in the gallbladder with one larger stone in the gallbladder neck, however, there was no evidence of choledocholithiasis in the CBD. The gastroenterologist felt that this imaging study confirmed the diagnosis of MS. General surgery was consulted and they scheduled a laparoscopic cholecystectomy for the following day. The patient’s EUS can be seen below in Figure 2.

The patient underwent a successful cholecystectomy on hospital day three. Surgical findings included a very short, wide, and firm cystic duct as well as the hepatic artery in very close proximity to the top of the CBD. The hepatic artery was adherent to the infundibulum of the gallbladder. This was speculated by the gastroenterologist to be due to inflammation from the gallstone’s compression. The gallbladder itself showed severely progressive diffuse inflammation as well.

**Figure 3:**
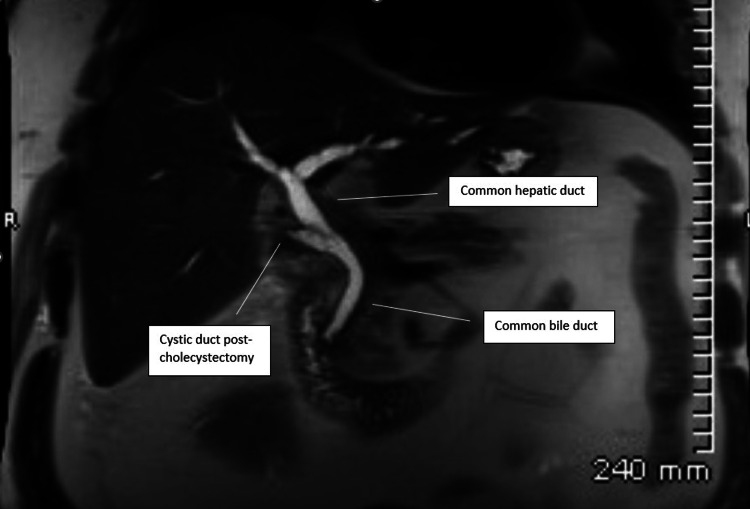
Patient's second MRCP completed on admission day five post-cholecystectomy. MRCP: magnetic resonance cholangiopancreatography

Two days later, on hospital day five, a second MRCP was performed due to the patient's persistently increased LFTs and a worsening jaundiced state post-cholecystectomy. Although the gallbladder was removed, there was new onset hepatic biliary dilatation with a 3 mm CBD stone. The patient’s second MRCP is pictured below in Figure 3.

On admission day seven the patient underwent ERCP with biliary sphincterotomy and all visible stones were removed. Over the course of the next two admission days, his LFTs gradually improved. The patient continued to remain stable and was discharged home with gastroenterology to follow up in two weeks.

## Discussion

Mirizzi syndrome is a rare complication of cholelithiasis involving external compression of the common hepatic duct from stones lodged in either the cystic duct or Hartmann’s pouch [1-3]. While this impingement typically remains external, if left untreated the chronic inflammation can lead to hepatic duct wall necrosis and subsequent cholecystobiliary fistula formation between the cystic duct and the CHD [7]. Epidemiology of MS varies dramatically between different studies, however, most research documents an overall incidence of around 1% in patients undergoing ERCP for cholecystitis [8]. Although extremely rare and thus difficult to diagnose, recognizing MS early through a combination of physical examination, labs, and imaging is important for successful treatment.

McSherry was the first physician to differentiate upon the classifications of patients with MS [1,9]. This system separated the condition into two different subtypes as follows: type I involves external obstruction of the common hepatic duct by a stone impacted in either the cystic duct or the Hartmann pouch, resulting in inflammation of the Calot triangle. Type II involves similar obstruction, however, it also includes erosion of the stone into the CHD from the cystic duct, forming a cholecystobiliary fistula [1,9]. While these definitions still hold true, the Csendes classification, which later expanded type II into three different subtypes based on the percentage of the CHD diameter which was obstructed, is the most widely accepted classification [10]. Csendes type II includes the presence of a cholecystobiliary fistula with <33% of the CHD, whereas type III and type IV obstruct 33-66% and >66%, respectively [10-12]. Table 1 describes the four classifications of MS. In the case previously described, our patient likely had type III or type IV MS due to obvious inflammation as well as severely elevated LFTs [10-12].

**Table 1:** Csendes classification of Mirizzi syndrome subtypes one through four. CHD: common hepatic duct

Mirizzi syndrome classification	Type I	Type II	Type III	Type IV
Pathophysiologic description	External compression of CHD without cholecystobiliary fistula formation	Cholecystobiliary fistula with <33% of the width of the CHD obstructed	Cholecystobiliary fistula with 33-66% of the width of the CHD obstructed	Cholecystobiliary fistula with >66% of the width of the CHD obstructed

Due to the pathophysiology of MS being similar to other causes of cholecystitis and biliary obstruction, the symptomatology is also rather nonspecific. Patient complaints classically resemble obstructive jaundice with symptoms such as nausea, vomiting, bloating, flatulence, and dull RUQ pain which radiates to the upper back. These symptoms often present in the evening after ingestion of a fatty meal [12]. Physical examination findings include scleral icterus, abdominal pain, and possible hepatomegaly. A positive Murphy’s sign is found in roughly 50% of patients [8]. Similar to physical presentation, labs are nonspecific and of little use in differentiating MS from other more common causes of obstructive jaundice such as choledocholithiasis and malignancy [13]. In one study, AST and ALT were found to be elevated in up to 98% of MS patients. ALP was elevated in 94% of patients, total bilirubin was elevated in 92%, and mild leukocytosis was seen in 73% due to the inflammatory processes of the obstruction [8]. Due to the low specificity, these symptoms, physical examination findings, and lab results are most useful in ruling out MS rather than aiding in its diagnosis.

Preoperative diagnosis of MS is of the utmost importance, as it is associated with decreased intraoperative complication rate, better outcomes, and avoidance of bile duct injury [1]. The initial imaging modality of choice for patients with symptoms of MS is a RUQ ultrasound. While ultrasound is very commonly used, diagnostic accuracy can be limited [14]. MRCP is generally the next step when right upper quadrant ultrasound reveals findings specific to obstructive jaundice such as atrophic gallbladder and dilated bile ducts. Due to its non-invasive imaging technique and diagnostic accuracy of up to 50%, MRCP is a highly effective and safe tool for working up patients suspected of MS [14]. Although the previously mentioned imaging modalities are first-line for evaluating patients with obstructive jaundice, the gold standard for diagnosing MS is the ERCP. With an average sensitivity of 76%, this technique yields superior visualization of the biliary tract and can clearly show external compression of the CHD by impacted gallstones. ERCP can also accurately diagnose cholecystobiliary fistulas in more advanced cases of this condition. Although there are clear advantages of ERCP, there is also an increased risk of complications, so caution should be taken when used in suspected MS [5,14]. As is referenced in the description of the aforementioned case, many of these imaging modalities were completed prior to coming to a consensus diagnosis.

The mainstay treatment of MS most often involves cholecystectomy or subtotal cholecystectomy [1]. Traditionally, open laparotomy is the technique of choice, largely due to relative safety compared to laparoscopy. Minimally invasive laparoscopy has many advantages such as shorter length of stay as well as reduced waste of resources, however, the conversion rate to open procedure is extremely high in MS, with some studies reporting it to be close to 80% [14]. This increased conversion rate has classically been attributed to the lack of preoperative diagnoses discussed previously [7]. Some experts recommend limiting laparoscopic approaches to patients with type I MS only, as further inflammation from the procedure could potentially exacerbate bile duct injury in patients with cholecystobiliary fistulas [15]. Complications from surgery are also quite common in patients undergoing treatment for MS, with one study stating the rate of complications being roughly 16%. The most common complications include bile duct injury, residual gallstones, and bile leakage [7]. Residual gallstones were found in the case described in this manuscript.

The patient described in this report underwent a relatively uncharacteristic diagnostic path with a EUS prior to ERCP secondary to temporary unavailability of the latter procedure. As is typical of MS, multiple different imaging modalities were utilized not only to diagnose that specific condition, but also to rule out all other more common causes of obstructive jaundice. Although less common than open laparotomy, his subsequent treatment with laparoscopic cholecystectomy yielded only partial relief of symptoms due to remnant stones migrating down to the CBD causing a subsequent choledocholithiasis. Once ERCP was able to be completed, complete resolution was possible when the final stones were removed from the CBD.

## Conclusions

Due to its relative infrequency and nonspecific symptomatology, MS can be difficult to diagnose leading to intraoperative complications or biliary duct injury. Here we presented a unique case of acute biliary colic and obstructive jaundice in a 32-year-old male. In a presentation such as this, MS should be considered in the differential. MRCP and ERCP are key in establishing a diagnosis, as seen in this case, with cholecystectomy being the treatment of choice. A careful classification of MS can determine the proper modality of cholecystectomy to ensure safe and effective treatment. This case provides an example of how MS can present without definitive radiological evidence and how proper treatment will lead to an uncomplicated clinical course. Further reporting of MS presentation and workup is needed to aid in prompt diagnosis and reduce possible intraoperative complications.
